# Deep learning based clinico-radiological model for paediatric brain tumor detection and subtype prediction

**DOI:** 10.37349/etat.2023.00159

**Published:** 2023-08-30

**Authors:** Abhishek Mahajan, Mayur Burrewar, Ujjwal Agarwal, Bharadwaj Kss, Apparao Mlv, Amrita Guha, Arpita Sahu, Amit Choudhari, Vivek Pawar, Vivek Punia, Sridhar Epari, Ayushi Sahay, Tejpal Gupta, Girish Chinnaswamy, Prakash Shetty, Aliasgar Moiyadi

**Affiliations:** University of Toronto, Canada; ^1^Clatterbridge Centre for Oncology NHS Foundation Trust, L7 8YA, Liverpool, UK; ^2^Department of Radiodiagnosis, Tata Memorial Hospital, Parel, Mumbai 400012, Maharashtra, India; ^3^Endimension Technology Pvt Ltd, Maharashtra, India; ^4^Department of Pathology, Tata Memorial Hospital, Parel, Mumbai 400012, India; ^5^Department of Paediatric Oncology, Tata Memorial Hospital, Parel, Mumbai 400012, India; ^6^Department of Surgical Oncology, Tata Memorial Hospital, Parel, Mumbai 400012, India

**Keywords:** Deep learning model, artificial intelligence, paediatric brain tumors, ependymoma, medulloblastoma, pilocytic astrocytoma, brainstem glioma

## Abstract

**Aim:**

Early diagnosis of paediatric brain tumors significantly improves the outcome. The aim is to study magnetic resonance imaging (MRI) features of paediatric brain tumors and to develop an automated segmentation (AS) tool which could segment and classify tumors using deep learning methods and compare with radiologist assessment.

**Methods:**

This study included 94 cases, of which 75 were diagnosed cases of ependymoma, medulloblastoma, brainstem glioma, and pilocytic astrocytoma and 19 were normal MRI brain cases. The data was randomized into training data, 64 cases; test data, 21 cases and validation data, 9 cases to devise a deep learning algorithm to segment the paediatric brain tumor. The sensitivity, specificity, positive predictive value (PPV), negative predictive value (NPV), and accuracy of the deep learning model were compared with radiologist’s findings. Performance evaluation of AS was done based on Dice score and Hausdorff95 distance.

**Results:**

Analysis of MRI semantic features was done with necrosis and haemorrhage as predicting features for ependymoma, diffusion restriction and cystic changes were predictors for medulloblastoma. The accuracy of detecting abnormalities was 90%, with a specificity of 100%. Further segmentation of the tumor into enhancing and non-enhancing components was done. The segmentation results for whole tumor (WT), enhancing tumor (ET), and non-enhancing tumor (NET) have been analyzed by Dice score and Hausdorff95 distance. The accuracy of prediction of all MRI features was compared with experienced radiologist’s findings. Substantial agreement observed between the classification by model and the radiologist’s given classification [K-0.695 (K is Cohen’s kappa score for interrater reliability)].

**Conclusions:**

The deep learning model had very high accuracy and specificity for predicting the magnetic resonance (MR) characteristics and close to 80% accuracy in predicting tumor type. This model can serve as a potential tool to make a timely and accurate diagnosis for radiologists not trained in neuroradiology.

## Introduction

Primary brain tumors are a heterogeneous group of benign as well as malignant tumors arising from the brain parenchyma and its surrounding structures. Brain tumors are the most common solid tumors in the paediatric age group and they are a leading cause of mortality and morbidity in children worldwide [1–3], exceeded only by leukaemia [3, 4]. According to most of the studies, the three most common types of paediatric brain tumors are astrocytoma, medulloblastoma, and ependymoma [1]. Overall survival in paediatric brain tumors varies with the type and grade of the tumor. It has been found that low-grade gliomas like pilocytic astrocytoma after gross total resection have 10-year progression-free survival of over 95% and have the best survival among paediatric brain tumors [5]. Children with non-disseminated medulloblastoma have an approximate 60% to 65% likelihood of survival for 5 years; however, the survival in disseminated tumors is less favourable, approximately 35% to 40% [5]. Ependymoma after gross total resection and radiotherapy is expected to have over 75% chance to survive for 5 years without any recurrence [5]. Brainstem glioma in children has a 3-year survival rate of only 5% to 15% even after treatment [6].

Early diagnosis and treatment of paediatric brain tumors significantly improve the outcomes [7]. To accurately diagnose paediatric brain tumors on imaging, specialized radiologists with experience in neuroradiology as well as in neuro-oncology are required. This niche requirement is often difficult to meet in many primary healthcare setups worldwide. Artificial intelligence (AI) is ever evolving tool in healthcare; especially in diagnostic oncology [8–11]. If accurate enough, AI-based tools can serve patients where specific skilled radiologists are not available. Recent advances in AI have made such diagnostic tools possible. Few machine learning-based algorithms have been developed in recent times [12]. Also, the role of machine learning in survival prediction and prognostication has been publicized in the field of neuro-oncology [13, 14]. The majority of brain tumor segmentation and radio genomics classification work has been published for glioblastoma in recent literature [15–17].

The purpose of this study was to study semantic and deep learning magnetic resonance imaging (MRI) features of paediatric brain tumors and to develop a deep learning based automated segmentation (AS) tool which could segment paediatric brain tumors and predict subtypes.

## Materials and methods

### Patient cohort

Approximately 2,500 patients registered between 2007–2020 at a tertiary cancer care institute were screened after clearance from the Institutional Ethics Committee. Cases with preoperative MRI in digital imaging and communications in medicine (DICOM) format were selected for the study. Clinical information obtained from electronic medical records. Clinical parameters assessed were age, sex, clinical features like seizures, gait ataxia, and cranial nerve palsy. Cases with histopathological diagnosis of ependymoma, medulloblastoma, and pilocytic astrocytoma were included in the study. Tumors for which biopsy is not routinely performed i.e., brainstem glioma and a few cases of pilocytic astrocytoma were also included, considering joint discussion of diagnosis by radiologist, radiation oncologist, and medical oncologist to be final. A total of 75 cases were selected. MRI semantic features were obtained for all 75 cases using available sequences. MRI semantic features included in this study are mentioned in Table 1.

**Table 1:** MRI semantic features

MR sequence	Intensity pattern	Intensity pattern	Intensity pattern
T1WI	Hyperintense	Isointense	Hypointense
T2WI	Hyperintense	Isointense	Hypointense
FLAIR	Hyperintense	Isointense	Hypointense
Haemorrhage	Present	Absent	-
Calcification	Present	Absent	-
DWI	Restriction	No restriction	-
Cyst	Present	Absent	-
Necrosis	Present	Absent	-
Tumor margins	Well defined	Ill defined	-
Enhancement pattern	Homogenous	Heterogeneous	-
Enhancement pattern	Mild (< 25%)	Moderate (25–75%)	Severe (> 75%)
Tumor location	Forebrain	Brainstem	Cerebellum and fourth ventricle
Laterality	Right	Left	Midline
Oedema	< Tumor volume	= Tumor volume	> Tumor volume
Midline shift	Present	Absent	-
Hydrocephalus	Present	Absent	-

MR: magnetic resonance; T1WI: T1 weighted image; FLAIR: fluid-attenuated inversion recovery; DWI: diffusion-weighted imaging; -: blank cell

MRI scans of these 75 cases (with brain tumors) and 19 other cases without any abnormality (i.e., without tumors) were also retrieved. Data was divided into training, validation, and test data sets and used to devise a deep learning-based algorithm to segment the paediatric brain tumor. These 94 cases were randomized and split into training data, 64 cases; test data, 21 cases, and validation data, 9 cases.

### Radiology review

**Figure 1:**
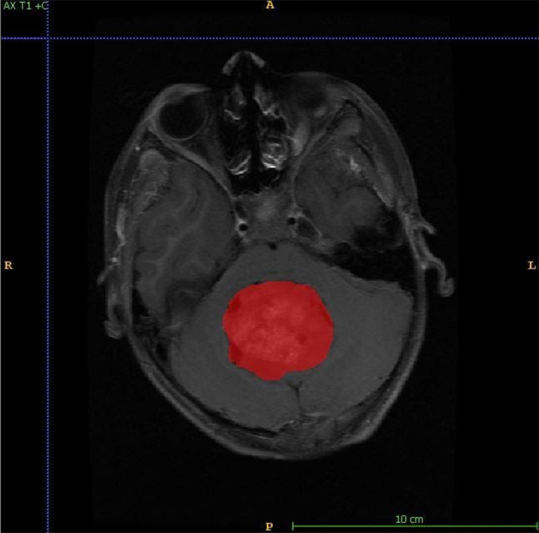
Figure shows annotation for tumor identification. A: anterior; P: posterior; R: right; L: left

**Figure 2:**
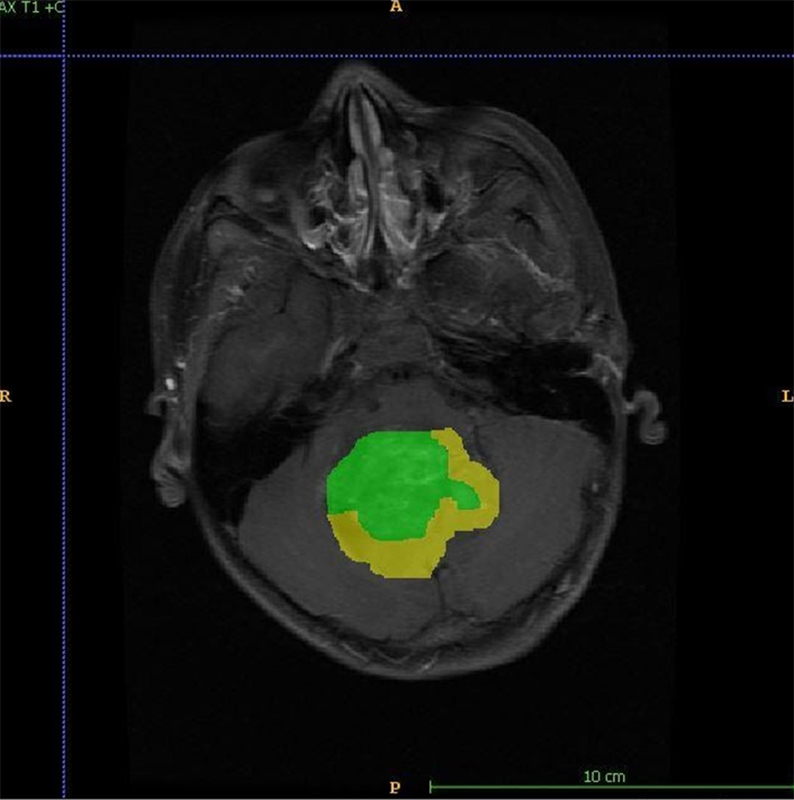
Figure shows annotation for enhancing (green) and non-enhancing (yellow) components of the tumor. A: anterior; P: posterior; R: right; L: left

**Figure 3:**
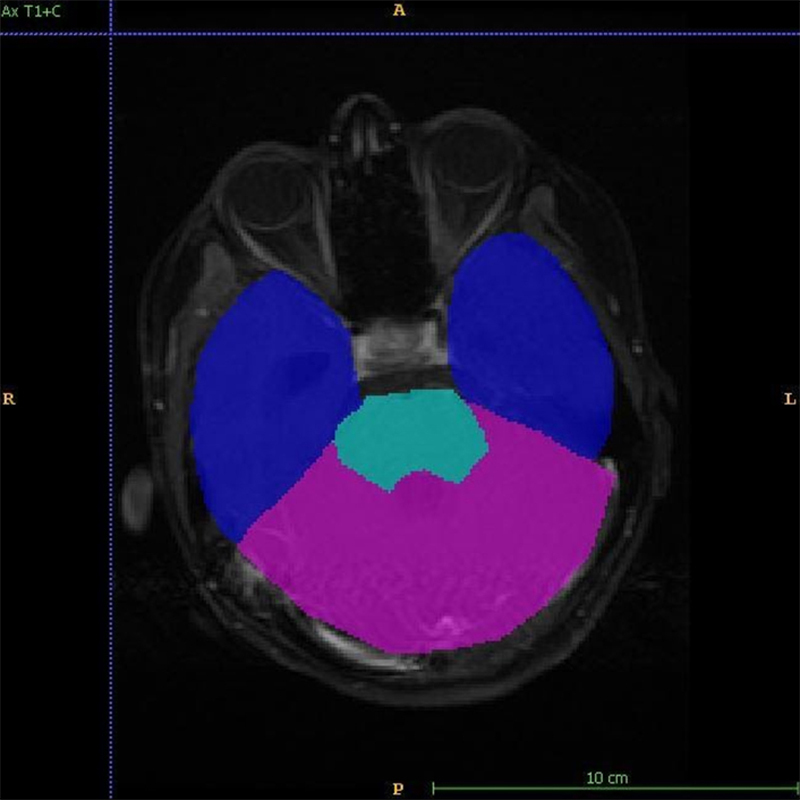
Figure shows annotations for training the model to identify the site of the tumor. Blue: forebrain; pink: cerebellum; aqua: brainstem; A: anterior; P: posterior; R: right; L: left

The MRI semantic features were detected by a radiologist with 12 years of experience in neuroimaging. In addition to routine sequences (T1, post-contrast T1, T2, FLAIR, and DWI), the gradient echo (GRE)/susceptibility-weighted imaging (SWI) sequences were evaluated for the detection of blooming within tumors. Isointensity was labelled when the tumor signal had similar intensity as that of grey matter, hypo intensity, and hyperintensity when the tumor signal had low and high intensity as compared to grey matter respectively. High signals on isotropic (DWI) images with corresponding low apparent diffusion coefficient (ADC) values were labelled as restricted diffusion. Enhancement was quantified as mild, moderate, and severe as compared to the entire tumor volume. The enhancement was labelled as heterogeneous or homogeneous. Training data is used to train the deep neural network, and validation data is used to check the network performance and fine-tune the network. Test data was used to assess the final accuracy of the trained model. In order to make models generalize better and create variations of the data, data augmentation was performed. For each patient, MRI data of size 240 × 240 × 155 was provided with FLAIR, T1, postcontrast T1, T2, and DWI sequences. The tumor was annotated on all slices in the post-contrast phase. Annotations for tumor identification were done as shown in Figure 1. Separate annotations were also done for enhancing and non-enhancing components of the tumor as shown in Figure 2. An annotation to identify a tumor site was shows in Figure 3. The annotations were verified by the same radiologist. The deep learning model was trained to identify the tumor, location, and then segmentation to identify the enhancing and non-enhancing component of the tumor. The deep learning-based model predicted focal T1 hyperintensity as a haemorrhage. For cyst and necrosis, the model classified cyst as structures that are T2 hyperintense and T1 hypointense and shows significant suppression (> 75%) and necrosis to structures which did not show significant suppression (< 25% or no suppression) on FLAIR. Deep learning based-model predicted features was showed in Table 2. For prediction of signal intensity on T1WI, T2WI, FLAIR, cyst, necrosis, diffusion restriction, tumor location, and enhancement, they followed similar identification criteria to an experienced radiologist.

**Table 2:** MR features used for deep learning-based model prediction

**MR sequence**	**Intensity pattern**	**Intensity pattern**	**Intensity pattern**
T1WI	Hyperintense	Isointense	Hypointense
T2WI	Hyperintense	Isointense	Hypointense
FLAIR	Hyperintense	Isointense	Hypointense
Haemorrhage	Present	Absent	-
DWI	Restriction	No restriction	-
Cyst	Present	Absent	-
Necrosis	Present	Absent	-
Enhancement pattern	Homogenous	Heterogeneous	-
Enhancement pattern	Mild (< 25%)	Moderate (25–75%)	Severe (> 75%)
Tumor location	Forebrain	Brainstem	Cerebellum and fourth ventricle

-: blank cell

### Development of deep learning algorithm and classification

**Figure 4:**
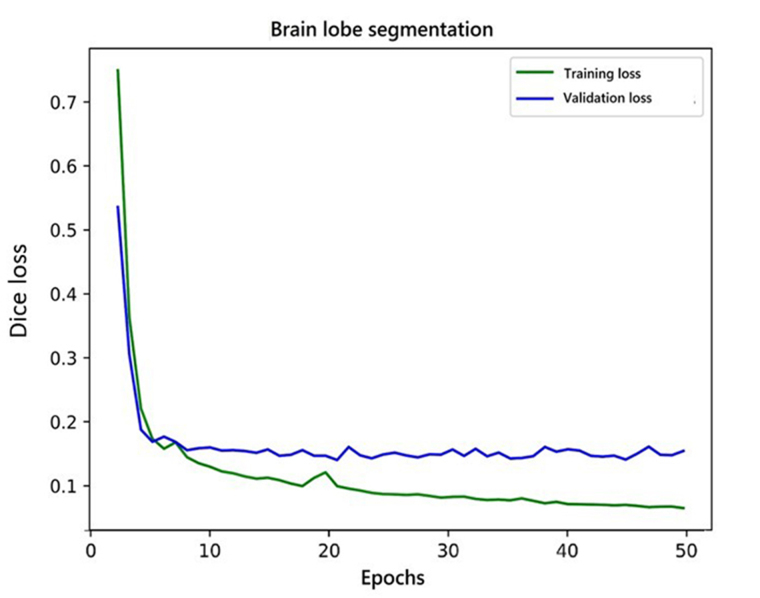
Training and validation curve for lobe segmentation network

**Figure 5:**
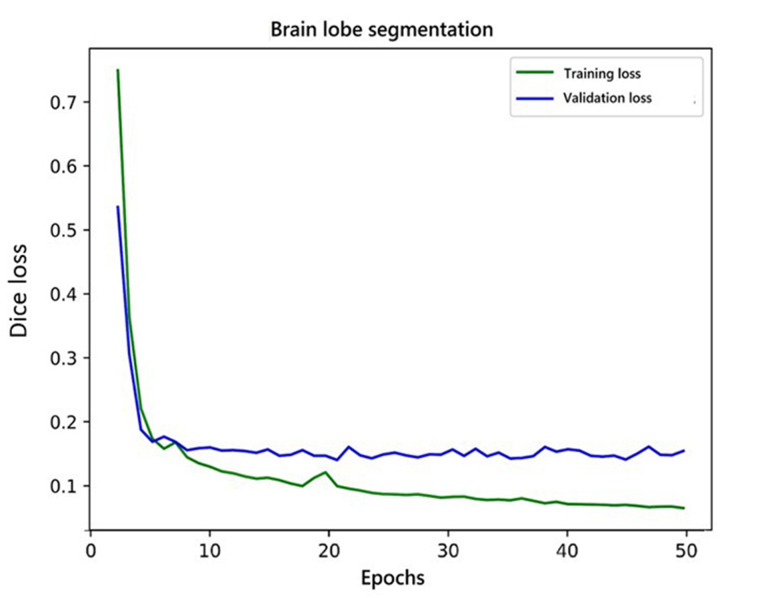
Training and validation curve for stage 1 network for tumor segmentation

**Figure 6:**
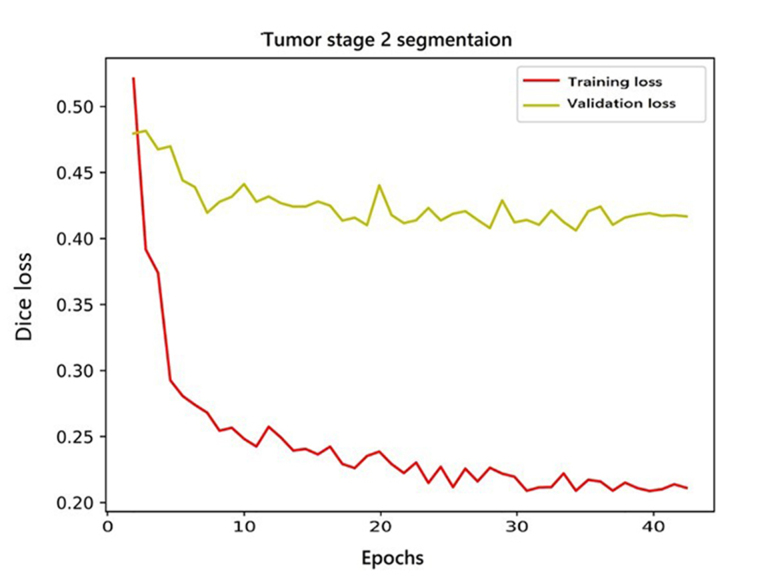
Training and validation curves of stage 2 network for tumor segmentation

The AI pipeline used two different models in a sequence. The first model performed lobe segmentation and the second model segmented tumors in the scan. The first model used a variation of three-dimensional (3D) U-NET [18] which takes a 256 × 256 × 24 voxel grid as input. Before feeding the data to the model, we clipped the voxel values from a range of 0 to 2,030. After this, the voxel values were standardized with a mean of 730 and a standard deviation (SD) of 361. The network gave three channels as output. These output labels were used to label each voxel of the 3D input as forebrain, brainstem, and cerebellum. The training and validation curve for the lobe segmentation network is shown in Figure 4. These predictions were later used to build the heuristic for post-processing of the results from the pipeline. The second model was a variation of 2D U-NET [18]. We processed the data at this stage in two steps. In the first step, scan slices were taken in the shape of 512 × 512 × 1 as input and predicted tumor segmentation. The second step took predictions of the first step and second channel of input along with the original slice and predicted two channels representing enhancing and non-enhancing components of the tumor. The training and validation curve for stage 1 and stage 2 network for tumor segmentation is shown in Figure 5 and Figure 6 respectively.

Before feeding the slices to the models, we clipped the values of the input from the 0 to 2,030 range and normalize the slice with a minimum of 162 and a maximum of 2,030. Dice loss [19] was used to train all the models. We trained the lobe segmentation model for 50 epochs and performed early stopping to avoid overfitting. For lobe segmentation, in the first stage, we trained the network for 35 epochs but the network failed to converge after that, so we stopped the training because the results were good enough to continue for the second stage network. The second stage network for tumor segmentation was trained for 40 epochs and it did not show any improvements after that.

### Statistical analysis

Statistical analysis was performed using Statistical Package for the Social Sciences (SPSS) version 21. All statistics were 2-sided, and a value of *P* < 0.05 was considered statistically significant. Performance evaluation of AS was done based on the basis of Dice Score and Hausdorff95 distance. The dice score is essentially a measure of overlap between two samples. This measure ranges from 0 to 1, where a dice coefficient of 1 denotes perfect and complete overlap. The Dice score normalizes the number of true positives to the average size of the two segmented areas [20]. The Hausdorff distance is a measure of similarity with respect to their position in metrix space [21]. The Hausdorff95 distance is the 95th quartile of the maximum overall surface distance between the predicted surface and the ground-true surface. The sensitivity, specificity, positive predictive value (PPV), negative predictive value (NPV), and accuracy of all deep learning-based predicted features were computed against radiologist-given MRI features. The predicted diagnosis by the model was compared with the radiologist’s diagnosis.

## Results

### MRI-based semantic features

Demographics revealed a median age of 8 years for ependymoma, 6 years for medulloblastoma, 9 years for pilocytic astrocytoma, and 7 years for brainstem glioma. Gait ataxia was a common presenting feature of brainstem glioma and medulloblastoma. Cranial nerve palsies were most commonly seen in patients with brainstem glioma.

Analysis showed 16 times more likelihood of tumors with necrosis to be ependymoma as opposed to tumors without necrosis. Tumors with haemorrhage were 4.9 times more likely to be ependymoma as opposed to tumors without haemorrhage. Tumors with diffusion restriction were 56.9 times more likely to be medulloblastoma as opposed to tumors without restricted diffusion on DWI. Tumors with cystic components were 25.3 times more likely to be medulloblastoma as opposed to tumors with no cystic components. Tumors without haemorrhage were 13.8 times more likely to be pilocytic astrocytoma as opposed to tumors with haemorrhage.

### Deep learning based AS model

For this part, a total of 94 cases (75 with tumors and 19 normal brain MRI scans) were included.

**Figure 7:**
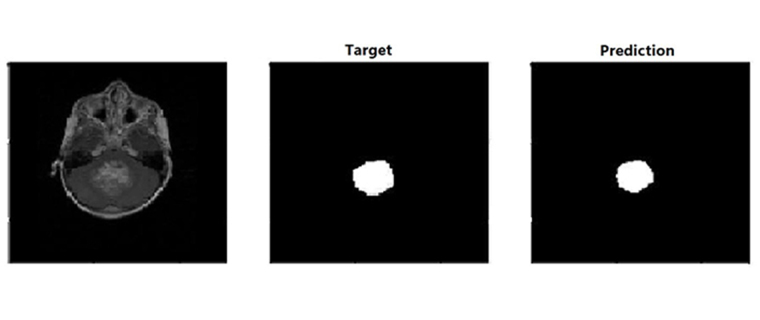
Target and actual prediction labels in a case of fourth ventricle brain tumor. Figure shows prediction label of the trained model

For each patient, MRI data of size 240 × 240 × 155 was used. We trained the model using 64 training cases, validated on 9, and tested on 21 cases. Out of 21 test cases (18 cases had tumors and 3 were normal), the AI-based model could identify tumors in 16 cases, and 2 cases were missed by the AI model. False positive cases were nil. The model didn’t predict any abnormality in normal scans or in normal slices in abnormal scans. Accuracy of detection of abnormality i.e., tumors in our trained mode was 0.90 i.e., 90%. The prediction label of a trained model is shown in Figure 7. The segmentation results for the prediction of tumor i.e., both enhancing and non-enhancing components were analyzed by Dice score and Hausdorff95 distance and mentioned in Table 3.

**Table 3:** Dice scores and Hausdorff95 distance of WT, ET, and NET for all datasets

**Dataset**	**Evaluation parameters**	**Dice score**			**Hausdorff95 distance**		
		**WT**	**ET**	**NET**	**WT**	**ET**	**NET**
Training data (64)	Mean	0.857	0.797	0.752	1.507	2.987	3.639
	SD	0.234	0.247	0.289	1.014	4.197	3.825
	25th quartile	0.870	0.751	0.693	0.870	1.000	1.000
	75th quartile	0.952	0.957	0.985	1.560	3.000	4.030
Validation data (9)	Mean	0.712	0.507	0.437	4.328	8.475	8.502
	SD	0.136	0.285	0.215	3.181	6.862	4.596
	25th quartile	0.593	0.351	0.354	0.593	2.236	5.029
	75th quartile	0.804	0.747	0.599	7.039	13.140	11.720
Test data (21)	Mean	0.606	0.586	0.312	8.130	3.412	20.430
	SD	0.342	0.370	0.384	17.500	3.145	21.260
	25th quartile	0.573	0.198	2.95e–8	0.573	1.207	6.140
	75th quartile	0.834	0.831	0.500	5.024	4.802	20.700

The numbers in parentheses are indicates number of examples used for training, validation and test

After training the model and segmentation, as mentioned in the materials and methodology section, all cases with tumors were subjected to heuristic rules over the deep learning-based segmentation model and features such as tumor signal intensity on T1WI, T2WI, FLAIR, enhancement pattern, cyst, necrosis, haemorrhage, and diffusion restriction were predicted for all cases. The diagnostic accuracy of feature prediction by the deep learning-based model as opposed to the radiologist detected MRI findings is shown in Table 4.

**Table 4:** Diagnostic accuracy of feature prediction by deep learning based model as opposed to radiologist detected MRI findings

**Predicted feature *n* = 70**		**Sensitivity**	**Specificity**	**PPV**	**NPV**	**Accuracy**
Hypointensity on T1WI		86.36%	0	93.44%	0	81.43%
Isointensity on T2WI		63.16%	84.62%	94.74%	34.37%	67.14%
Hyperintensity on FLAIR		85.71%	61.9%	60%	86.67%	71.4%
Haemorrhage		54.17%	78.26%	56.62%	76.6%	70%
Cyst		79.07%	59.26%	75.56%	64%	71.4%
Necrosis		52.94%	83.02%	50%	84.62%	75.71%
Enhancement heterogeneity		81.36%	72.73%	94.12%	42.11%	80%
Enhancement quantification	Mild	50%	92.86%	82.35%	73.58%	75.71%
	Moderate	61.1%	71.15%	42.31%	84.09%	68.57%
	Severe	83.3%	84.78%	74.07%	90.70%	84.29%
Diffusion restriction		53.33%	96.77%	94.12%	68.16%	75.4%

*n*: total number of abnormal cases which were picked by AS model and on which the mentioned features are predicted by AS model

### Deep learning-based prediction of tumor type

The deep learning-based model classified the tumors into one of the four types according to the predicted anatomical site and predicted features. The sensitivity, specificity, PPV, NPV, and accuracy of the deep learning-based model given diagnosis as well as diagnosis given by imaging findings by experienced radiologist were computed against the final diagnosis of all cases and mentioned in Table 5. The inter-rater reliability between predicted diagnosis by model and diagnosis by the radiologist was calculated by Kappa’s agreement coefficient. The measure of Agreement-Kappa value was 0.695 for prediction of diagnosis by deep learning model as compared to the diagnosis given on imaging by an experienced radiologist. Statistically, it shows substantial agreement (0.61–0.80).

**Table 5:** Diagnostic accuracy of deep learning-based model predicted diagnosis and experienced radiologist gave a diagnosis

**Tumor group**		**Sensitivity**	**Specificity**	**PPV**	**NPV**	**Accuracy**
Brainstem glioma	Predicted by model	88.24%	96.23%	88.24%	96.24%	94.29%
	Diagnosis by radiologist	100%	98.11%	94.44%	100%	98.57%
Ependymoma	Predicted by model	64.29%	96.43%	81.82%	91.53%	90%
	Diagnosis by radiologist	92.86%	96.43%	86.67%	98.17%	98.18%
Medulloblastoma	Predicted by model	61.9%	100%	100%	85.96%	88.57%
	Diagnosis by radiologist	95.24%	100%	100%	98%	98.57%
Pilocytic astrocytoma	Predicted by model	100%	78.85%	62.07%	100%	84.29%
	Diagnosis by radiologist	88%	97.62%	94.12%	95.35%	95%

## Discussion

AI has recently made substantial strides in perception (the interpretation of sensory information), allowing machines to better represent and interpret complex data. Deep learning is a subset of machine learning that is based on a neural network structure inspired by the human brain. These neural networks learn discriminative features from data automatically, giving them the ability to approximate very complex nonlinear relationships. Recent methods based on deep convolutional neural networks have outperformed all traditional machine learning methods in various domains like medical image segmentation, image classification, object detection, and tracking. In the first part of the present study, clinical features and MRI based semantic features were evaluated. A total of 75 cases were included. In the second part of the study deep learning based algorithm was developed and tested to detect the abnormality in the MRI scan and segmentation into enhancing and non-enhancing components and tumor subtypes. A total of 94 cases were included in this part of the study.

The median age at diagnosis of brainstem glioma was 7 years and more common in males. A study by Hong et al. [6] revealed similar demographic features [22]. A study by Hong et al. [6] had 84% brainstem glioma cases with cranial nerve palsy involvement and 67% brainstem glioma cases with cerebellar ataxia as compared to 83.3% brainstem glioma cases with cranial nerve involvement and 94.4 % brainstem glioma cases with gait ataxia in the present study. In the present study, pilocytic astrocytoma was the second most common of these four tumors to have cranial nerve involvement (47.4%) and medulloblastoma was the second most common of these four tumors to have gait ataxia (55.6%). Univariate analysis of all tumor groups for clinical features is mentioned in the following Table 6. *P*-values of combined univariate analysis of all tumor groups for MRI semantic features are mentioned in Table 7.

**Table 6:** A *P*-value of combined univariate analysis of all tumor groups for clinical features

**Clinical features**		**Ependymoma, *n* = 14**	**Medulloblastoma, *n* = 21**	**Pilocytic astrocytoma, *n* = 19**	**Brainstem glioma, *n* = 21**	***P*-value**
Seizures	Yes	0	1 (5.6%)	4 (21.1%)	2 (11.1%)	0.264
	No	11 (100.0%)	17 (94.4%)	15 (78.9%)	16 (88.9%)	
Gait ataxia	Yes	3 (27.3%)	10 (55.6%)	5 (26.3%)	17 (94.4%)	< 0.001
	No	8 (72.7%)	8 (44.4%)	14 (73.7%)	1 (5.6%)	
Cranial nerve palsy	Yes	3 (27.3%)	6 (33.3%)	6 (33.3%)	15 (83.3%)	0.006
	No	8 (72.7%)	12 (66.7%)	10 (52.6%)	3 (16.7%)	

The brackets in the table show the percentage of statistics

**Table 7:** *P*-value of combined univariate analysis of all tumors groups for MRI semantic features

**MRI features**		**Ependy-moma, *n* = 14**	**Medullo-Blastoma, *n* = 21**	**Pilocytic astrocytoma, *n* = 19**	**Brainstem glioma, *n* = 21**	***P*-value**
T1WI	Hypointense	12 (85.7%)	21 (100.0%)	17 (89.5%)	20 (95.2%)	0.336
	Isointense	2 (14.3%)	0	2 (10.5%)	1 (4.8%)	
T2WI	Hyperintense	2 (14.3%)	0	6 (31.6%)	6 (28.6%)	0.047
	Hypointense	1 (7.1%)	0	0	0	
	Isointense	11 (78.6%)	21 (100.0%)	13 (68.4%)	15 (71.4%)	
FLAIR	Hyperintense	11 (78.6%)	1 (4.8%)	12 (63.2%)	6 (28.6%)	< 0.001
	Isointense	3 (21.4%)	20 (95.2%)	7 (36.8%)	15 (71.4%)	
Enhancement pattern	Homogenous	1 (7.1%)	5 (23.8%)	5 (26.3%)	0	0.053
	Heterogeneous	13 (92.9%)	16 (76.2%)	14 (73.7%)	21 (100.0%)	
Enhancement pattern	Mild < 25%	3 (21.4%)	6 (28.6%)	5 (26.3%)	17 (81.0%)	0.001
	Moderate 25–75%	5 (35.7%)	5 (23.8%)	6 (31.6%)	4 (19.0%)	
	Severe > 75%	6 (42.9%)	10 (47.6%)	8 (42.1%)	0	
Haemorrhage	No	5 (35.7%)	13 (61.9%)	18 (94.7%)	16 (76.2%)	0.003
	Yes	9 (64.3%)	8 (38.1%)	1 (5.3%)	5 (23.8%)	
Calcification	No	9 (64.3%)	20 (95.2%)	19 (100.0%)	21 (100.0%)	< 0.001
	Yes	5 (35.7%)	1 (4.8%)	0	0	
DWI	No restriction	10 (76.9%)	1 (5.9%)	13 (76.5%)	14 (70%)	< 0.001
	Restriction	3 (23.1%)	16 (94.1%)	4 (23.5%)	6 (30%)	
Cyst	No	4 (28.6%)	2 (9.5%)	5 (26.3%)	20 (95.2%)	< 0.001
	Yes	10 (71.4%)	19 (90.5%)	14 (73.7%)	1 (4.8%)	
Necrosis	No	4 (28.6%)	20 (95.2%)	19 (100.0%)	13 (61.9%)	< 0.001
	Yes	10 (71.4%)	1 (4.8%)	0	8 (38.1%)	
Tumor margins	Well defined	6 (42.9%)	19 (90.5%)	15 (78.9%)	3 (14.3%)	< 0.001
	Ill defined	8 (57.1%)	2 (9.5%)	4 (21.1%)	18 (85.7%)	
Tumor location	Forebrain	6 (42.9%)	0	13 (68.4%)	0	< 0.001
	Brainstem	0	0	0	21 (100.0%)	
	Cerebellum and fourth ventricle	8 (57.1%)	21 (100.0%)	6 (31.6%)	0	
Laterality	Right	2 (14.3%)	1 (4.8%)	4 (21.1%)	3 (14.3%)	0.225
	Left	4 (28.6%)	1 (4.8%)	4 (21.1%)	2 (9.5%)	
	Midline	8 (57.1%)	19 (90.5%)	11 (57.9%)	16 (76.2%)	
Oedema	No	4 (28.6%)	15 (71.4%)	7 (36.8%)	5 (23.8%)	0.048
	Less than tumor volume	9 (64.3%)	6 (28.6%)	10 (52.6%)	16 (76.2%)	
	Equal to tumor volume	0	0	1 (5.3%)	0	
	More than tumor volume	1 (7.1%)	0	1 (5.3%)	0	
Midline shift	No	12 (85.7%)	21 (100.0%)	17 (89.5%)	21 (100.0%)	0.133
	Yes	2 (14.3%)	0	2 (10.5%)	0	
Hydrocephalus	Absent	5 (35.7%)	0	8 (42.1%)	12 (57.1%)	0.001
	Present	9 (64.3%)	21 (100.0%)	11 (57.9%)	9 (42.9%)	

The median age at diagnosis of ependymoma was found to be 8 years and more common in males. The mean age of ependymoma was 4.5 years according to a study by Duc et al. [23]. Ependymoma were mostly hypointense on T1WI, isointense on T2WI, and hyperintense on FLAIR. Infratentorial location was seen in 57.1% of cases. Almost all tumors (92.9%) showed heterogeneous enhancement. Hemorrhage was present in 64.3% of cases, higher than the rest of the tumor groups. Calcification was present in 35.7% of cases, relatively more common than in other groups. Diffusion restriction was present in 23.1 % of cases. Necrosis was seen in 71.4% of cases, higher than other groups. Cystic changes were seen in 71.4% of cases. Hydrocephalus was seen in 64.3 % of cases at presentation. A study by Mangalore et al. [24] with 41 cases had hydrocephalus in 34% of cases, calcification in 78% of cases and heterogeneous enhancement in all cases. However, the study by Mangalore et al. [24] mainly considered computerized tomography (CT) imaging findings as only 8 cases had baseline MRI imaging [24]. Multivariate analysis showed 16 times more odds of a tumor with necrosis having a final diagnosis of ependymoma than tumors without necrosis. It was also found that tumors with hemorrhage had 4.9 times more odds to have a final diagnosis of ependymoma than tumors without hemorrhage. Median age at diagnosis of medulloblastoma was found to be 6 years and more common in males. Median age of medulloblastoma was 9 years according to a study by Arora et al. [25]. Medulloblastoma was hypointense on T1WI and mostly isointense on T2WI and FLAIR. Midline tumor seen in 90.5 % of cases. Heterogeneous enhancement was seen in 76.2% of cases, whereas 23.8% cases showed homogeneous enhancement. Hemorrhage was present in 38.1% of cases, less than ependymoma. Calcification was seen in only 4.8% i.e., in only one case. Restricted diffusion was observed in 94.1% of cases. Cystic changes were seen in 90.5% of cases. All cases had hydrocephalus at presentation. Multivariate analysis showed 56.9 times more odds of a tumor showing restricted diffusion on DWI to have a final diagnosis of medulloblastoma than tumors not showing diffusion restriction. It was also observed that tumors with cystic changes had 25.3 times more odds to have a final diagnosis of medulloblastoma than tumors without cystic changes. Comparative analysis of a few MRI findings of medulloblastoma in the present study with two previously published studies is given in following Table 8 [26, 27].

**Table 8:** Comparative analysis of MRI sematic features of medulloblastoma in the present study with previous studies by Hussain et al. [26] and Yeom et al. [27]

**Medulloblastoma**		**Present study *n* = 21**	**Study by Hussain et al. [26] *n* = 29**	**Study by Yeom et al. [27] *n* = 38**
Tumor location	Midline	90.5%	89.7%	82%
	Others i.e., lateral	9.5%	10.3%	18%
Tumor margins	Well defined	90.5%	51.7%	82%
	Ill defined	9.5%	48.3%	18%
Enhancement	Homogeneous	23.8%	24.1%	-
	Heterogeneous	76.2%	75.9%	
Cyst		90.5%	69%	≤ 1 cm (55%) > 1 cm (24%)
Necrosis		4.8%	20.7%	-
Peritumoral oedema		28.6%	-	50%

-: blank cell

The median age at diagnosis of pilocytic astrocytoma was found to be 9 years and more common in males. The median age of pilocytic astrocytoma was 10 years according to a study by Arora et al. [25]. Pilocytic astrocytoma was mostly hypointense on T1WI, isointense on T2WI, and hyperintense on FLAIR. Tumor was seen to be localized in forebrain in 68.4% of cases whereas 31.6% of cases were infratentorial. Heterogeneous enhancement was seen in 73.7% cases, whereas 26.3% cases showed homogeneous enhancement. Hemorrhage was present in 5.1%, less than ependymoma and medulloblastoma. Calcification was not detected in any case. Restricted diffusion observed in 23.5% of cases. Cystic changes were seen in 73.7% cases. Around 57.9% of cases had hydrocephalus at presentation. Multivariate analysis showed 13.8 times less odds of a tumor with hemorrhage to have a final diagnosis of pilocytic astrocytoma than tumors without hemorrhage.

Brainstem glioma was mostly hypointense on T1WI and isointense on T2WI and FLAIR. Heterogeneous enhancement was seen in all the cases. Haemorrhage was present in 23.8% of cases, less than ependymoma and medulloblastoma. Restricted diffusion was observed in 30% of cases. Necrosis was seen in 38.1% of cases, however, cystic changes were rarely seen (only in one case). Around 42.9% of cases had hydrocephalus at presentation. A multivariate analysis of a few significant univariate MRI features of ependymoma, medulloblastoma, and pilocytic astrocytoma is shown in Table 9.

**Table 9:** Multivariate analysis of significant univariate MRI features

**MRI features**		***P*-value**	**Odds ratio**
Ependymoma	Necrosis Present	0.001	16.1
	Haemorrhage Present	0.024	4.9
Medulloblastoma	Diffusion restriction Present	< 0.001	56.9
	Cyst Present	0.006	25.3
Pilocytic astrocytoma	Haemorrhage Absent	0.015	13.8

Imaging findings were congruent with published literature by Poretti et al. [28], Plaza et al. [29], and Camacho et al. [30]. Survival analysis was not statistically significant in the present study, however findings concurrent with literature i.e., best for pilocytic astrocytoma and worst for brainstem glioma. A deep learning-based algorithm was developed and tested to detect the abnormality in the MRI scan and segmentation into enhancing and non-enhancing components. The sensitivity and specificity of this model for detecting abnormalities are 0.88 and 1 respectively (accuracy is 0.9 i.e., 90%). For the classification of paediatric brain tumors, this model showed the highest accuracy in the detection of brainstem glioma i.e., 94.2%. The accuracy of detection of pilocytic astrocytoma by this model was least i.e., 84.29%. The accuracy of detection of ependymoma and medulloblastoma was 90% and 88.57% respectively. Quon et al. [31], developed a deep learning model in a study with 617 children, which had overall classification accuracy of 92% and sensitivity of 0.96 and specificity of 1 for tumor detection [31]. However, it was a multi-institutional study. Their model was most accurate at predicting diffuse midline glioma followed by pilocytic astrocytoma and medulloblastoma [31]. Ependymoma prediction was the least accurate [31]. They considered the interpretation by four radiologists for comparison, out of which the model showed greater accuracy than two radiologists [31]. Another multi-institutional study of the deep learning-based model with 288 patients for tumor classification by Zhou et al. [32] showed an accuracy of 85% for medulloblastoma *versus* non-medulloblastoma, the accuracy of 80% for ependymoma *versus* non-ependymoma, and an accuracy of 88% for pilocytic astrocytoma *versus* non-pilocytic astrocytoma [32] as shown in Table 10. It had significantly higher accuracy than the average qualitative expert MR imaging review [32].

**Table 10:** Comparison of diagnostic accuracy of deep learning-based approach in the present study with a study by Zhou et al. [32]

**Tumor group**	**Present study (*n* = 94)**	**Study by Zhou et al. [32] (*n* = 288)**
Brainstem glioma	94.2%	Not included
Medulloblastoma	88.57%	85%
Ependymoma	90%	80%
Pilocytic astrocytoma	84.29%	88%

Our study shows that the prediction of tumor types by experienced radiologists was more accurate than deep learning model prediction. This primarily is related to the fact that a smaller number of cases were included in this study for training and validation purposes. The accuracy of the model can be increased further in a greater number of cases. Nevertheless, the model may form the basis of molecular genetics prediction by deep learning of the most common paediatric brain tumors.

In conclusion AI has the potential in localizing paediatric brain tumors and feature detection as well as diagnosis. The proposed deep learning-based model had very high accuracy and specificity for predicting the individual MR characteristics and close to 80% accuracy in predicting tumor type. This model can serve as a potential tool to aid to make timely and accurate diagnosis for radiologist not specialized/trained in neuroradiology and neuro-oncology. Nevertheless, the model may also form the basis of a multi-omics (clinical radiological and pathological) model for predicting molecular genetics by deep learning/machine learning.

## Abbreviations

AI: artificial intelligence
AS: automated segmentation
DWI: diffusion-weighted imaging
ET: enhancing tumor
FLAIR: fluid-attenuated inversion recovery
MR: magnetic resonance
MRI: magnetic resonance imaging
NET: non-enhancing tumor
SD: standard deviation
T1WI: T1 weighted image
WT: whole tumor

## Declarations

### Availability of data and materials

The datasets generated in this study are available on request to the corresponding author, Abhishek Mahajan (drabhishek.mahajan@yahoo.in; abhiradiology@gmail.com).

### Copyright

© The Author(s) 2023.

## References

[1] Madhavan R, Kannabiran BP, Nithya AM, Kani J, Balasubramaniam P, Shanmugakumar S (2016). Pediatric brain tumors: an analysis of 5 years of data from a tertiary cancer care center, India. Indian J Cancer.

[2] AlRayahi J, Zapotocky M, Ramaswamy V, Hanagandi P, Branson H, Mubarak W (2018). Pediatric brain tumor genetics: what radiologists need to know. Radiographics.

[3] Lacayo A, Farmer PM (1991). Brain tumors in children: a review. Ann Clin Lab Sci.

[4] Panigrahy A, Blüml S (2009). Neuroimaging of pediatric brain tumors: from basic to advanced magnetic resonance imaging (MRI). J Child Neurol.

[5] Packer RJ (2008). Childhood brain tumors: accomplishments and ongoing challenges. J Child Neurol.

[6] Hong S, Kim IH, Wang KC (2005). Outcome and prognostic factors of childhood diffuse brainstem glioma. Cancer Res Treat.

[7] Ali ZA, Habib RM, Fotoh SA (2020). Role of magnetic resonance imaging in diagnosis of pediatric posterior fossa tumors. Menoufia Medical J.

[8] Mahajan A, Vaidya T, Gupta A, Rane S, Gupta S (2019). Artificial intelligence in healthcare in developing nations: the beginning of a transformative journey. Cancer Res Stat Treat.

[9] Bothra M, Mahajan A (2020). Mining artificial intelligence in oncology: Tata Memorial Hospital journey. Cancer Res Stat Treat.

[10] Davatzikos C, Barnholtz-Sloan JS, Bakas S, Colen R, Mahajan A, Quintero CB (2020). AI-based prognostic imaging biomarkers for precision neuro-oncology: the ReSPOND consortium. Neuro Oncol.

[11] Cherian Kurian N, Sethi A, Reddy Konduru A, Mahajan A, Rane SU (2021). A 2021 update on cancer image analytics with deep learning. WIREs Data Mining Knowl Discov.

[12] Akbari H, Mohan S, Garcia JA, Kazerooni AF, Sako C, Bakas S (2021). NIMG-22. Prediction of glioblastoma cellular infiltration and recurrence using machine learning and multi-parametric mri analysis: results from the multi-institutional respond consortium. Neuro-Oncology.

[13] Bakas S, Reyes M, Jakab A, Bauer S, Rempfler M, Crimi A Identifying the best machine learning algorithms for brain tumor segmentation, progression assessment, and overall survival prediction in the BRATS challenge.

[14] Baid U, Rane SU, Talbar S, Gupta S, Thakur MH, Moiyadi A (2020). Overall survival prediction in glioblastoma with radiomic features using machine learning. Front Comput Neurosci.

[15] Baid U, Ghodasara S, Mohan S, Bilello M, Calabrese E, Colak E RSNA-ASNR-MICCAI BraTS 2021 Benchmarkon brain tumor segmentation and radiogenomic classification.

[16] Mehta R, Filos A, Baid U, Sako C, McKinley R, Rebsamen M QU-BraTS: MICCAI BraTS 2020 challenge on quantifying uncertainty in brain tumor segmentation--analysis of ranking metrics and benchmarking results.

[17] Baid U, Talbar S, Rane S, Gupta S, Thakur MH, Moiyadi A (2020). A novel approach for fully automatic intra-tumor segmentation with 3D U-Net architecture for gliomas. Front Comput Neurosci.

[18] Rudie JD, Weiss DA, Colby JB, Rauschecker AM, Laguna B, Braunstein S (2021). Three-dimensional U-Net convolutional neural network for detection and segmentation of intracranial metastases. Radiol Artif Intell.

[19] Zhang J, Shen X, Zhuo T, Zhou H Brain tumor segmentation based on refined fully convolutional neural networks with a hierarchical dice loss.

[20] Sudre CH, Li W, Vercauteren T, Ourselin S, Jorge Cardoso M, Cardoso MJ, Arbel T, Carneiro G, Syeda-Mahmood T, Tavares JMRS, Moradi M (2017). Generalised dice overlap as a deep learning loss function for highly unbalanced segmentations. Deep learning in medical image analysis and multimodal learning for clinical decision support.

[21] Piramuthu S The Hausdorff distance measure for feature selection in learning applications.

[22] Zimmerman RA (1996). Neuroimaging of pediatric brain stem diseases other than brain stem glioma. Pediatr Neurosurg.

[23] Duc NM, Huy HQ (2019). Magnetic resonance imaging features of common posterior fossa brain tumors in children: a preliminary vietnamese study. Open Access Maced J Med Sci.

[24] Mangalore S, Aryan S, Prasad C, Santosh V (2015). Imaging characteristics of supratentorial ependymomas: study on a large single institutional cohort with histopathological correlation. Asian J Neurosurg.

[25] Arora RS, Alston RD, Eden TO, Estlin EJ, Moran A, Birch JM (2009). Age–incidence patterns of primary CNS tumors in children, adolescents, and adults in England. Neuro-oncology.

[26] Hussain IZ, Mohd Zaki F, Mukari SA, Md Pauzi SH, Loh CK, Alias H (2020). Correlation between MRI characteristics of medulloblastoma with histopathological subtypes and 2-year survival. Indian J Radiol Imaging.

[27] Yeom KW, Mobley BC, Lober RM, Andre JB, Partap S, Vogel H (2013). Distinctive MRI features of pediatric medulloblastoma subtypes. AJR Am J Roentgenol.

[28] Poretti A, Meoded A, Huisman TA (2012). Neuroimaging of pediatric posterior fossa tumors including review of the literature. J Magn Reson Imaging.

[29] Plaza MJ, Borja MJ, Altman N, Saigal G (2013). Conventional and advanced MRI features of pediatric intracranial tumors: posterior fossa and suprasellar tumors. AJR Am J Roentgenol.

[30] Camacho AC, Chaljub G, Uribe T, Patterson JT, Swischuk LE (2007). MR imaging of pediatric posterior fossa tumors. Contemp Diagn Radiol.

[31] Quon JL, Bala W, Chen LC, Wright J, Kim LH, Han M (2020). Deep learning for pediatric posterior fossa tumor detection and classification: a multi-institutional study. AJNR Am J Neuroradiol.

[32] Zhou H, Hu R, Tang O, Hu C, Tang L, Chang K (2020). Automatic machine learning to differentiate pediatric posterior fossa tumors on routine MR imaging. AJNR Am J Neuroradiol.

